# Recent Advances in Cotton Genomics

**DOI:** 10.1155/2008/742304

**Published:** 2008-01-23

**Authors:** Hong-Bin Zhang, Yaning Li, Baohua Wang, Peng W. Chee

**Affiliations:** ^1^Department of Soil and Crop Sciences, Texas A&M University, College Station, TX 77843, USA; ^2^Department of Plant Pathology, Biological Control Center of Plant Diseases and Plant Pests of Hebei Province, Agricultural University of Hebei, Baoding 071001, China; ^3^Molecular Cotton Breeding Laboratory, University of Georgia, Tifton, GA 31793, USA; ^4^School of Life Sciences, Nantong University, Nantong 226007, China

## Abstract

Genome research promises to promote continued and enhanced plant genetic improvement. As a world's leading crop and a model system for studies of many biological processes, genomics research of cottons has advanced rapidly in the past few years. This article presents a comprehensive review on the recent advances of cotton genomics research. The reviewed areas include DNA markers, genetic maps, mapped genes and QTLs, ESTs, microarrays, gene expression profiling, BAC and BIBAC libraries, physical mapping, genome sequencing, and applications of genomic tools in cotton breeding. Analysis of the current status of each of the genome research areas suggests that the areas of physical mapping, QTL fine mapping, genome sequencing, nonfiber and nonovule EST development, gene expression profiling, and association studies between gene expression and fiber trait performance should be emphasized currently and in near future to accelerate utilization of the genomics research achievements for enhancing cotton genetic improvement.

## 1. INTRODUCTION

**Figure 1:**
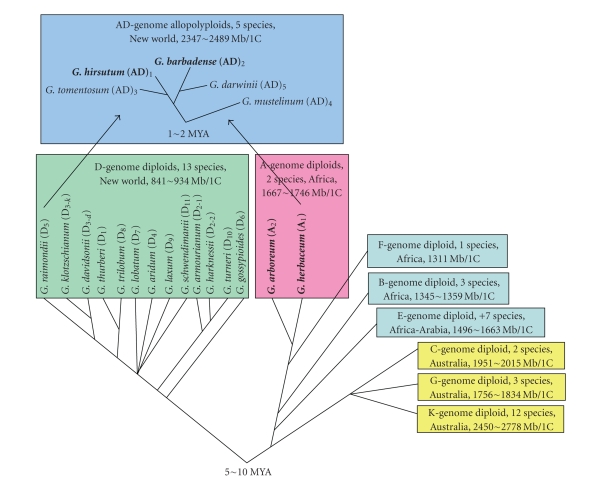
Phylogeny and evolution of *Gossypium* species. The phylogenetic data is from Wendel and Cronn [2], the genome sizes are from Hendrix and Stewart [3], and genomic designations follow Endrizzi et al. [4] and Percival [5]. The species in bold face are cultivated. MYA: million years ago.

**Figure 2:**
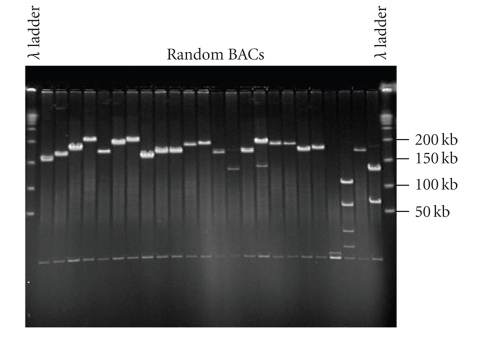
BACs randomly selected from the TM-1/*Eco* RI BAC library (see Table 3; C. Scheuring and H.-B. Zhang, unpublished). BAC DNA was isolated, digested with *Not* I, and run on a pulsed-field gel.

Cottons (*Gossypium spp.*) belong to the genus *Gossypium* of the family Malvaceae. *Gossypium* consists of 45–50 species, with 40–45 being diploids (2*n* = 26) and 5 being allotetraploids (2*n* = 52). The species are grouped into eight genome groups, designated A through G and K, on the basis of chromosome pairing affinities [1]. At the tetraploid level, there are five species, designated (AD)_1_ through (AD)_5_ for their genome constitutions. Phylogenetic analyses clustered the diploid species of *Gossypium* into two major lineages, including the 13 D-genome species lineage and the 30∼32 A-, B-, E-, F-, C-, G-, and K-genome species lineage, and the polyploid species into one lineage, that is, the 5 AD-genome species lineage (Figure 1; [2]).

Of the *Gossypium* species, four are cultivated in agriculture, including two allotetraploids (*G. hirsutum* and *G. barbadense*) and two diploids (*G. herbaceum* and *G. arboreum*). *Gossypium hirsutum,* also known as Upland cotton, Long Staple Cotton, or Mexican Cotton, produces over 90% of the world’s cotton; *G. barbadense*, also known as Sea Island Cotton, Extra Long Staple Cotton, American Pima, or Egyptian Cotton, contributes 8% of the world’s cotton; and *G. herbaceum*, also known as Levant Cotton, and *G. arboreum*, also known as Tree Cotton, together provide 2% of the world’s cotton.

Cottons are not only a world’s leading textile fiber and oilseed crop, but also a crop that is of significance for foil energy and bioengergy production. Although cottons are native to tropics and subtropics naturally, including the Americas, Africa and Asia, they are cultivated in nearly 100 countries. India, China, USA, and Pakistan are the top four cotton growing countries, accounting for approximately 2/3 of the world’s cotton (http://www.ers.usda.gov/Briefing/Cotton/trade.htm). According to the Food and Agriculture Organization (FAO) of the United Nations (http://www.fao.org), the cotton planting area reached about 35 million hectares and the total world’s cotton production had a record of about 23 million metric tones in 2004/2005. Cotton products include fibers and seeds that have a variety of uses. Cotton fibers sustain one of the world’s largest industries, the textile industry, for wearing apparel, home furnishings, and medical supplies, whereas cottonseeds are widely used for food oil, animal feeds, and industrial materials (such as soap). Cottonseed oil is ranked fifth in production and consumption volume among all vegetable oils in the past decades, accounting for 8% of the world’s vegetable oil consumption. The business stimulated by cotton is hundreds of billion dollars in the world. In the USA alone, for instance, the annual cotton business revenue exceeds $120 billion (Agricultural Statistics Board 1999; National Cotton Council of America, http://www.cotton.org/news/releases/2003/cotton-trade.cfm). Moreover, nearly a billion barrels of petroleum worldwide are used in every year to synthesize artificial “synthetic” fibers. Further improvement of cotton fibers in yield and quality will replace or significantly reduce the consumption of fossil oil for synthetic fiber production, thus being saved for energy production. Finally, cottonseed oil, the main by-product of cotton fiber production, could be potentially used as biofuel.

In addition to their economic importance, cottons are an excellent model system for several important biological studies, including plant genome size evolution, plant polyploidization and single-celled biological processes. The genomes of angiosperm plants vary over 1000 folds in size, ranging from 100 to >100,000 Mb/1C (haploid) [6]. It has long been recognized that polyploidy is a common, prominent, ongoing, and dynamic process of genome organization, function diversification, and evolution in angiosperms [7]. The genomes of most angiosperms are thought to have incurred one or more polyploidization events during evolution [8]. Studies have demonstrated that genome doubling has also been significant in the evolutionary history of all vertebrates and in many other eukaryotes [9–12]. It is estimatedthat about 70% of the flowering plant species are polyploids. For instance, of the world-leading field, forage, horticultural, and environmental crops, many are contributed by polyploid species, such as cotton, wheat, soybean, potatoes, canola, sugarcane, *Brassica*, oats, peanut, tobacco, rose, coffee, and banana. Therefore, studies of both genome size evolution and polyploidization have long attracted the interests of scientists in different disciplines. Nevertheless, much remains to be learned. Examples include impacts of polyploidization on genome size, genome organization, gene duplication and function, and gene family evolution; the role of transposable elements in structural and regulatory gene evolution and gene functions; and mechanisms and functional significance of rapid genome changes.

Cottons have several advantages over other polyploid complexes for plant genome size and polyploidization studies. First, the genome sizes of 37 of the 45∼50 *Gossypium* species, including all eight genomes and polyploidy species, have been determined and shown to vary extremely significantly ([3]; Figure 1). At the diploid level, the genome sizes vary by three folds, ranging from 885 Mb/1C in the D-genome species to 2,572 Mb/1C in the K-genome species. Within each lineage, the genome sizes vary most in the A+F+B+E+C+G+K lineage, ranging from 1,311 to 2,778 Mb/1C with a difference of 1,467 Mb (110.2%); second in the D-genome lineage, ranging from 841 to 934 Mb/1C with a difference of 93 Mb (10.5%); and least in the polyploidy lineage, ranging from 2,347 to 2,489 Mb/1C with a difference of 142 Mb (5.9%). Variations were also observed within a species. For instance, within G. *hirsutum*, the variation (*n* = 5) was from 2,347 to 2,489 Mb/1C, differing by 142 Mb (5.9%) while within *G. arboreum*, the variation (*n* = 5) was from 1,677 to 1,746 Mb/1C, differing by 69 Mb (4.0%).

Second, the evolutionary history of the allotetraploid species of *Gossypium* has been established (Figure 1), especially for the two cultivated AD-genome cottons, *G. hirsutum* and *G. barbadense,* and their closely related diploid progenitors, *G. herbaceum* (A_1_), *G. arboreum* (A_2_), *G. raimondii* (D_5_), and *G. gossypioides* (D_6_). The A-genome species are African-Asian in origin, whereas the D-genome species are endemic to the New World subtropics, primarily Mexico. Following the transoceanic dispersal of an A-genome taxon to the New World, hybridization between the immigrant A-genome taxon and a local D-genome taxon led to the origin and evolution of the New World allopolyploids (AD-genome) [13, 14]. Subsequent to the polyploidization event, the allopolyploids radiated into three sublineages [15], among which included are the world’s commercially most important species, *G. hirsutum* and *G. barbadense*. Studies showed that the A subgenome of the AD-genome-cultivated cottons is the most closely related to the genome of the extant diploid *G. herbaceum* (A_1_) [16]; the D subgenome of the AD-genome-cultivated cottons is the most closely related to the genome of the extant diploid, *G. raimondii* (D_5_) or *G. gossypioides* (D_6_) [13]; and the cytoplasm of the AD-genome-cultivated cottons is the most closely related to that of the extant diploids *G. herbaceum* (A_1_) and *G. arboreum* (A_2_) [14, 17]. Sequence analysis and paleontological record suggest that the A-genome and the D-genome groups diverged from a common ancestor 5–10 million years ago, and that the two diverged diploid genomes became reunited in a common nucleus to form the polyploid cottons, via allopolyploidization, in the mid-Pleistocene, or 1-2 million years ago [14, 15, 18, 19].

Finally, as in the wheat polyploid complex, cottons have a long history of research at the cytological level. A wealth of cytogenetic stocks has been developed, including artificially synthesized AD-genome polyploids between the A-genome and D-genome diploid species [20] as well as individual chromosome addition and substitution lines [21]. These cytogenetic stocks are unique and valuable not only for cotton genetics research, but also for deciphering the ramifications of polyploidization on genome organization, function, and evolution.

Cotton fiber is an excellent single-celled model system for studies of many single-celled biological processes, particularly cell expansion and cellulose biosynthesis. Cotton fibers are unicellular, unbranched, simple trichomes that differentiate from the protoderm of developing seeds. There are probably over one-half million quasi-synchronously elongating fibers in each boll or ovary. Although all plant cells extend to some degree during development and differentiation, cotton fibers can reach up to 5.0 cm in length in some genotypes, being among the longest cells. Therefore, they offer a unique opportunity to study cell expansion at the single cell level. Cellulose is a major component of the cell walls of all higher plants, constituting perhaps the largest component of plant biomass, with an estimated annual world production of 100 million metric tons. The fiber cell wall of cottons consists of >90% cellulose. Therefore, cotton fiber cells have long been used as a model system to study cellulose biosynthesis [22] that is the basis for biomass-based bioenergy production.

## 2. ADVANCES IN COTTON GENOMICS RESEARCH

Genome research has been demonstrated to be promising for continued and enhanced crop plant genetic improvement. Therefore, efforts have been made in cotton genome research, especially development of genomic resources and tools for basic and applied genetics, genomics, and breeding research. These resources and tools include different types of DNA markers such as restriction fragment length polymorphism (RFLP), randomly amplified polymorphic DNA (RAPD), amplified fragment length polymorphism (AFLP), resistance gene analogs (RGA), sequence-related amplified polymorphism (SRAP), simple sequence repeat (SSR) or microsatellites, DNA marker-based genetic linkage maps, QTLs and genes for the traits important to agriculture, expressed sequence tags (ESTs), arrayed large-insert bacterial artificial chromosome (BAC) and plant-transformation-competent binary BAC (BIBAC) libraries, and genome-wide, cDNA-, or unigene EST-based microarrays. Efforts are also being made to develop the genome-wide, BAC/BIBAC-based integrated physical and genetic maps, and sequence the genomes of the key cotton species. However, compared with other major crops, such as rice, maize, and soybean, the genome research of cottons is far behind, mainly due to the limited funds allocated to the species. Summarized below are the major advances achieved recently in cotton genomics research.

### 2.1. DNA markers and molecular linkage maps

Genetic maps constructed in the *Gossypium* species and the types of markers used are listed in Table 1. As in most plant species, the early application of DNA markers in cotton genomic research has been in the form of RFLPs. It is, therefore, not surprising that the first molecular linkage map of the *Gossypium* species was constructed from an interspecific *G. hirsutum* × *G. barbadense* F_2_ population based on RFLPs [23]. The map contained 705 loci that assembled into 41 linkage groups and spanned 4,675 cM. This map later was further advanced by Rong et al. [24] that comprised 2,584 loci at 1.74-cM intervals and covered all 13 homeologous chromosomes of the allotetraploid cottons, representing the most complete genetic map of the *Gossypium* to date. Many of the DNA probes of the map were also mapped in crosses of the D-genome diploid species *G. trilobum* × *G. raimondii* [24] and the A-genome diploid species *G. arboreum* × *G. herbaceum* [16]. Detailed comparative analysis of the relationship of gene orders between the tetraploid AD-subgenomes with the maps of the A and D diploid genomes has revealed intriguing insights on the organization, transmission and evolution of the *Gossypium* genomes.

Because RFLPs are labor-intensive and require large amounts of DNA, tedious blot hybridization and autoradiographic methods, polymerase chain reaction (PCR)-based DNA marker methods have come into vogue. Several types of PCR-based DNA markers have been utilized in cotton genome research. Methods, such as RAPD, AFLP, RGA, and SRAP, offer an excellent opportunity to scan enormous numbers of DNA loci rapidly, often targeting the DNA elements that are rapidly-evolving and therefore, are more likely to contain loci differing among genotypes. Kohel et al. [25] constructed a genetic map based on a population derived from an interspecific cross between Texas Marker-1 (TM-1) (*G. hirsutum*) and 3–79 (*G. barbadense*) in which a total of 355 DNA markers (216 RFLPs and 139 RAPDs) were assembled into 50 linkage groups, covering 4,766 cM. Brubaker and Brown [26] presented the first AFLP genetic linkage map for the *Gossypium* G-genome that was constructed from an interspecific *G. nelsonii* × *G. australe* population. The AFLP genetic linkage maps were used to identify G-genome chromosome-specific molecular markers, which, in turn, were used to track the fidelity and frequency of *G. australe* chromosome transmission in a *G. hirsutum* × *G. australe* hexaploid bridging family.

Advent of SSR or microsatellite markers has brought a new, user-friendly, and highly polymorphic class of genetic markers for cotton. The latter feature is especially useful to the cultivated Upland cotton due to its low intraspecific polymorphism. SSRs are PCR-based markers, usually codominant, well dispersed throughout the genome, easily shared between labs via flanking primer sequences, and well portable from one population to another [84]. Reddy et al. [85] suggested that the total pool of SSRs present in the cotton genome is sufficiently abundant to satisfy the requirements of extensive genome mapping and marker-assisted selection (MAS). Liu et al. [86] reported the assignment of SSRs to cotton chromosomes by making use of aneuploid stocks. SSRs have been widely employed in genetic diversity analyses of cotton [87–90] and several genetic linkage maps based mostly on SSRs have now been developed [37–41].

Several methods have been pursued to develop SSR markers in cottons, including analysis of SSR-enriched small-insert genomic DNA libraries [29, 85, 86, 91], SSR mining from ESTs (see below; [35, 38, 39, 92], and large-insert BAC derivation by end sequence analysis [36] or SSR-containing BAC subcloning as described by Lichtenzveig et al. [93]. Currently, a total of approximately 5,484 SSRs have been developed in cotton ([94]; http://www.cottonmarker.org).

The development of a large number of ESTs (see below) provides a good source of PCR-based primers for targeting SSRs [92, 95, 96]. Taliercio et al. [97] sequenced ESTs representing a variety of tissues and treatments with SSRs identified among the ESTs. Their results indicated that these SSRs could potentially map the genes represented by the ESTs. Guo et al. [98] examined the transferability of 207 *G. arboreum*-derived EST-SSR primer pairs among 25 different diploid accessions from 23 species representing 7*Gossypium * genomes. Their results demonstrated that the transferability of EST-SSR markers among these diploid species could assist the introgression of genes into cultivated cotton species especially by molecular tagging of the important genes existing in these diploid species. Guo et al. [40] also developed 2,218 EST-SSRs, with 1,554 from *G. raimondii*-derived ESTs and 754 from *G. hirsutum*-derived ESTs. By integrating these new EST-SSRs to enhance the genetic map constructed by Han et al. [39], the present SSR-based genetic map consists of 1,790 loci in 26 linkage groups and covers 3,425.8 cM with an average distance between markers of 1.91 cM. This SSR-based high-density map contains 71.96% functional marker loci of which 87.11% are EST-SSR loci.

DNA sequences derived from clone end sequencing of BAC libraries provide yet another resource for SSR marker development. In addition to the uses as genetic markers, SSRs developed from BAC-end sequences provide the possibility to efficiently integrate the genetic and physical maps of cotton. Frelichowski et al. [36] developed 1,316 PCR primer pairs to flank SSR motif sequences from 2,603 new BAC-end genomic sequences developed from *G. hirsutum* Acala “Maxxa.” An interspecific recombinant inbred population was used to map 433 marker loci in 46 linkage groups with a total genetic distance of 2,126.3 cM and an average distance between loci of 4.9 cM which covered approximately 45% of the cotton genome.

To overcome the paucity of a particular type of DNA markers, genetic maps were developed by incorporating different classes of markers. For example, Lacape et al. [28] constructed a combined RFLP-SSR-AFLP map based on an interspecific *G. hirsutum* × *G. barbadense* backcross population of 75 BC_1_ plants. The map consists of 888 loci that ordered into 37 linkage groups and spanning 4,400 cM. This map was updated, mostly with new SSR markers, to contain 1,160 loci that spanned 5,519 cM with an average distance between loci of 4.8 cM [29]. Mei et al. [27] developed a genetic map using an interspecific *G. hirsutum* and *G. barbadense* F_2_ population that contained 392 genetic loci, including AFLPs, SSRs, and RFLPs, and mapped into 42 linkage groups that spanned 3,287 cM, thus covering approximately 70% of the cotton genome. Lin et al. [33] constructed a linkage map of tetraploid cotton using SRAPs, SSRs, and RAPDs to screen an interspecific *G. hirsutum* × *G. barbadense* F_2_ population. A total of 566 loci were assembled into 41 linkages that covered 5,141.8 cM with a mean interlocus space of 9.08 cM. He et al. [34] constructed a more detailed cotton map with this same F_2_ population [33] using SSRs, SRAP, RAPD, and retrotransposon-microsatellite amplified polymorphisms (REMAPs). One thousand twenty nine loci were mapped to 26 linkage groups that extended for 5,472.3 cM with an average distance between loci of 5.32 cM. The linkage groups of the genetic maps have been assigned to their corresponding chromosomes by using the available cotton aneuploid stocks [21, 23] and fluorescent in situ hybridization using mapped genetic marker-containing BACs as probes [99].

### 2.2. Gene and QTL mapping

Although molecular linkage maps have contributed greatly to our understanding of the evolution and organization of the cotton genomes, a primary purpose of the map construction is to provide a common point of reference for locating the genes affecting qualitative and quantitative traits. DNA markers that are associated with genes conferring important agronomic traits that are costly or laborious to measure will provide a less costly and yet more dependable means of selection for identifying desirable progenies in breeding programs.

**Mapping qualitative traits**

Qualitative or simple Mendelian inherited traits are traits of individuals that differ as to kind and not of degree, typically controlled by single genes and the phenotypic variation falls into discrete classes in the segregating progenies. Over 200 qualitative traits have been identified in either the diploid (*G. arboreum* and *G. herbaceum*) or tetraploid (mostly in *G. hirsutum* and *G. barbadense*) species [1]. Examples of such traits include leaf shape, pollen color, leaf color, lint color, pubescent, bract shape, and so on. Because many qualitative traits are either morphological mutants that have arisen through spontaneous mutation, irradiation, or from natural variation between species in interspecific hybrids, they have little utility in crop improvement. Consequently, there have been little efforts in mapping qualitative traits onto the molecular genetic map. Qualitative traits that have been mapped using molecular markers were recently summarized in [105]. Many of these traits were mapped not as the main objective but as a tool for aligning the various linkage groups to chromosomes assigned by the classical map. Noteworthy exceptions include those that are related to agricultural productivity and quality of cotton and can be broadly grouped into four categories; genes for leaf shape, fiber development, resistant to disease and insect pests, and fertility restoration [105].

**Mapping quantitative traits**

Quantitative traits are traits of individuals that differ as to degree and not of kind, typically considered as interactions of multiple loci, tend to exhibit continuous variation in a segregating population, and are readily subjected to variation of environments. With the increased availability of DNA markers for use in cotton genetic map construction in the last ten years, activities in identifying and locating quantitative trait loci (QTLs) have blossomed. QTLs that have been identified in cotton include yield and yield components, fiber quality, plant architecture, resistance to diseases such as bacterial blight and *Verticillium* wilt, resistance to pests like root-knot nematode, and flowering date. A list of QTLs mapped in cotton is presented in Table 2.

Several noteworthy findings have come out of QTL mapping in cotton. First, in tetraploid cottons, although the D-subgenome was derived from an ancestor that does not produce spinnable fibers, many QTLs influencing fiber quality traits were detected on the D-subgenome [106]. For example, Jiang et al. [45] pointed out that D-subgenome QTLs may partly explain the fact that domestication and breeding of tetraploid cottons has resulted in fiber with a higher quality than those achieved by parallel improvement of the A-genome diploid cottons which produce spinnable fibers. The merger of the A- and D-genomes in tetraploid cottons, where each genome has a different evolutionary history, may have offered unique avenues for phenotypic response to selection. Second, numerous studies have shown that QTLs occur in clusters genetically in the cotton genome [27, 46, 55, 56, 76, 106]. Ulloa et al. [56] suggested the possible existence of highly recombined regions in the cotton genome with abundant putative genes. QTL clusters might exert their multiple functions to compensate for a numerical deficiency, expanding their roles in cotton growth and development [76]. Finally, the position and effect of QTLs for fiber quality are not comparable in different populations and environments evaluated [60, 106]. This suggests that QTL studies conducted thus far have detected only a small number of loci for fiber growth and development and that additional QTLs remain to be discovered [58, 59]. Furthermore, because quantitative traits are readily subjected to variation of environments, mapping efforts of these traits needs to be pursued in multiple environments including years and locations.

### 2.3. BAC and BIBAC resources

Large-insert BAC and BIBAC libraries have been demonstrated essential and desirable for advanced genomics and genetics research [107–111]. Because of their low-level chimerism, readily amenability to high-throughput purification of cloned insert DNA, and high stability in the host cell [83, 112, 113], BACs and BIBACs have quickly assumed a central position in genome research. BAC and BIBAC libraries have widely been used in many research areas of genomics and molecular biology, including whole-genome or chromosome physical mapping [110, 114–128], large-scale genome sequencing [129–133], positional cloning of genes and QTLs (for review, see [134]), isolation and characterization of structural and regulatory genes [135, 136], long-range genome analysis [135, 136], organization and evolution of multigene families [136], and cytologically physical mapping [137].

BAC libraries have been developed for a number of species, including plants, animals, insects, and microbes and made available to the public (http://hbz7.tamu.edu; http://bacpac.chori.org; http://www.genome.clemson.edu). To facilitate cotton genome research, BAC and BIBAC libraries have been developed for several genotypes of Upland cotton, *G. hirsutum* (Table 3). As of May 1, 2007, at least six BAC and BIBAC libraries have been developed and made available to the public. These libraries were constructed from five genotypes of Upland cotton, including Tamcot HQ95, Auburn 623, TM-1, Maxxa, and 0-613-2R, in four BAC vectors and one Agrobacterum-mediated, plant-transformation-competent BIBAC vector with three restriction enzymes. The libraries have average insert sizes ranging from 93 to 175 kb and each have a genome coverage ranging from 2.3 to 8.3x genome equivalents, collectively covering >21x haploid genomes of the polyploid cotton (Figure 2). Moreover, BAC libraries have also been constructed for several other *Gossypium* species, including *G. barbadense* (Pima S6), *G. arboreum* (AKA8401), *G. raimindii*, and *G. longicalyx* (A.H. Paterson, pers. communication). These BAC and BIBAC libraries provide resources essential for advanced genomics and genetics research of cottons.

### 2.4. ESTs, microarrays, and gene expression profiling

**ESTs**

Cloning and sequencing of expressed gene sequence tags (ESTs) by single sequencing pass from one or both ends of cDNA clones have been widely used to rapidly discover and characterize genes in a large-scale and high-throughput manner. As have been done in many other plant and animal species of biological and/or economical importance, significant efforts have been made to generate ESTs in cottons. As of April 27, 2007, 281,233 ESTs have been available for the *Gossypium* species in GenBank (Table 4; http://www.ncbi.nlm.nih.gov/dbEST). Of these ESTs, 178,177 were from the polyploid cultivated cottons with 177,154 (63.0%) from *G. hirsutum* and 1,023 (<1.0%) from *G. barbadnese* while 103,056 were from the related diploid species with 39,232 (13.9%) from *G. arboreum* (A_2_), 63,577 (22.6%) from *G. raimondii* (D_5_), and 247 (<1.0%) from *G. herbaceum* (A_1_). This number of cotton ESTs, compared with that of five years ago, has been significantly increased, due to several large EST projects funded [100–104]. Nevertheless, when compared with those of other major crop species such as rice, maize, wheat, and soybean, the number of the cotton ESTs is very low, only being about one-forth of those of rice, maize, or wheat (Table 4).

Table 5 summaries 247,979 ESTs of cottons published [100, 102–104]. These ESTs were collectively generated from 32 cDNA libraries constructed from mRNA isolated from 18 genotypes of three species, *G. hirsutum*, *G. arboretum,* and *G. raimondii*, by one-pass sequencing of cDNA clones from one (3′ or 5′ end) or both ends. They were generated from 12 different organs, including developing fibers, seedlings, buds, bolls, ovules, roots, hypocotyls, immature embryos, leaves, stems, and cotyledons. Some of the ESTs were generated from plants growing under biotic or abiotic stress conditions such as drought, chilling, and pathogens. By analyzing approximately 185,000 ESTs from both fibers/ovules (124,299 ESTs) and nonfiber/ovule tissues (60,899 ESTs) of *G. hirsutum*, *G. arboreum* and *G. raimondii*, Udall et al. [102] obtained 51,107 unigenes. A few months later, Yang et al. [103] analyzed their 32,789 ESTs generated from −3- to +3-dpa fibers of Upland cotton cv. TM-1, along with 211,397 cotton ESTs downloaded from GenBank (as of April 2006), resulting in 55,673 unigenes and updating The Institute of Genomic Research Cotton Gene Index version 6 (CGI6) into CGI7 (http://www.tigr.org). The unigene EST number may provide a reasonable estimation about the number of expressed genes in the cotton genomes. Of the unigene set, those derived from fibers or fiber-bearing ovules suggest the number of genes potentially involved in fiber development and genetic complexity of fiber traits.

**Figure 3:**
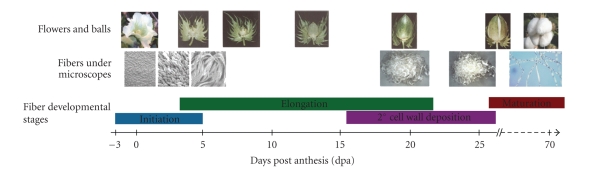
Cotton fiber development and corresponding morphogenesis stages (according to [138,139]). The initiation stage is characterized by the enlargement and protrusion of epidermal cells from the ovular surface; during the elongation stage the cells expend in polar directions with a rate of >2 mm/day; during the secondary cell wall deposition stage celluloses are synthesized rapidly until the fibers contain ∼90% of cellulose; and at the maturation stages minerals accumulate in the fibers and the fibers dehydrate.

A predominant feature of the cotton EST set is the significant preference of their tissue sources for fiber or fiber-bearing ovules than other organs. Of the 247,979 ESTs listed in Table 5, 187,080 (75.4%) were from developing fibers or fiber-bearing ovules while only 60,899 (24.6%) were from nonfiber and nonovule organs. Within each of the two categories, fiber/fiber-bearing ovules and nonfiber/ovule organs, there is also a significant bias in the number of ESTs. Cotton fiber development is classified into four clearly characterized, but overlapping stages, including fiber initiation (−3- to 5 dpa), elongation (5–25 dpa), secondary cell wall deposition (15–45 dpa), and maturation/dehydration (45–70 dpa) (see Figure 3). All of the 187,080 fiber ESTs were generated from the fibers or fiber-bearing ovules collected from the first three stages with 43.6% from the initiation stage, 46.5% from the elongation stage, and 5.7% from the secondary cell wall deposition stage. It is apparent that the number of fiber ESTs from the secondary cell wall deposition stage is much smaller than that of either initiation or elongation stage. Although the initiation and elongation stages are of significance for the number of fibers per seed and fiber length, the secondary cell wall deposition stage is crucial to fiber strength. Of the 60,899 nonfiber/nonovule ESTs, 66.0% were from seedlings, 14.2% from stems, and 2.4% from roots.

The cotton ESTs have been used in several aspects, including development of genome-wide cotton microarrays (see below), mining of SSRs (see above) and study of polyploidization. The development of the significant numbers of ESTs from the cultivated tetraploid cotton, *G. hirsutum* [(AADD)_1_], and its closely related diploid species, *G. arboreum* (A_1_A_1_) and *G. raimondii* (D_5_D_5_) (see Table 5) made it possible to compare the transcriptomes among the three species. Udall et al. [102] comparatively analyzed 31,424, 68,732, and 69,853 ESTs derived from *G. arboreum*, *G. raimondii*, and *G. hirsutum*, respectively. Although the comparison was significantly affected by the tissue sources and developmental status, they identified the putative homoeologs among the four genomes, A, D, A_1_, and D_5_. This information is useful for our understanding of how the cotton genomes function and evolve during the courses of speciation, domestication, plant breeding, and polyploidization.

**Microarray**

Microarray has been a technology that is widely used in many aspects of genomics research, including gene discovery, gene expression profiling, mutation assay, high-throughput genetic mapping, gene expression mapping (eQTL mapping), and comparative genome analysis. It involves robotically printing tens of thousands of cDNA amplicons or gene-specific long (70 mers) oligonucleotides as array elements on a chemically-coated glass slide, followed by hybridizing the array with one or more fluorescent-labeled cDNA or cRNA targets derived from mRNA isolated from particular tissues, organs, or cells. Therefore, it allows the simultaneous monitoring of the expression/activities of all genes arrayed on the array in a single hybridization experiment. To facilitate cotton genomics research, microarrays have been developed from the cotton ESTs (Table 5) in several laboratories worldwide.

**Figure 4:**
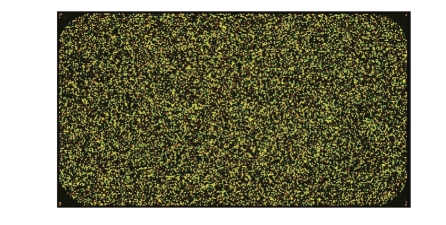
Comparative analysis of the expression of fiber genes in 10-dpa fibers between *G. hirsutum* and *G. barbadense* (M. Goebel, M. Alabady, C.W. Smith, T. A. Wilkins and H.-B. Zhang, unpublished). The cotton fiber microarrays are available in the laboratory of T. A. Wilkins, Texas Tech University (Tex, USA). One of the four arrays printed on a single slide is shown.

The first batch of cotton microarrays was fabricated from 70-mers oligos designed from the 7–10 dpa fiber nonredundant (NR) or unigene ESTs of *G. arboreum* (Table 5) ([100]; http://cottongenomecenter.ucdavis.edu/microarrays.asp). Each microarray consists of 12,227 elements corresponding to 12,227 NR fiber ESTs, with a duplicate of each element. Using the microarrays, Arpat et al. [100] compared the expression of the genes between 10-dpa fibers at elongation or primary cell wall synthesis stage and 24-dpa fibers at secondary cell wall disposition stage (see Figure 3). The expression of fiber genes was found to change dynamically from elongation or primary cell wall to secondary cell wall biogenesis, with 2,553 of the fiber genes being significantly downregulated and 81 being significantly upregulated. This result suggests that the expression of fiber genes is stage-specific or cell expansion-associated. Annotation of the genes upregulated in the secondary cell wall synthesis relative to the primary cell wall biogenesis showed that most of the genes felt in three major functional categories, energy/metabolism, cell structure, organization and biogenesis, and cytoskeleton. This finding is consistent with the fact of massive cellulose synthesis and cell wall biogenesis during this stage. The fiber gene microarrays have been updated recently by incorporating nearly 10,000 gene elements designed from the fiber and ovary ESTs of the tetraploid cultivated cotton, *G. hirsutum* (Table 5; T.A. Wilkins, pers. communication). The current fiber microarrays each slide consist of four duplicated arrays with 22,406 60-mers oligo elements per array and a duplicate of each element (see, e.g., Figure 4). The new version of fiber gene arrays covers 100% of the fiber ESTs of diploid cotton and 65% of the fiber ESTs of the tetraploid cultivated cotton, *G. hirsutum* that are available in GenBank, thus representing the most comprehensive coverage of the cotton fiber genes. The elements are printed on a slide in a randomized manner instead of the conventional ordered manner. The fabrication of four duplicated arrays per slide and randomized printing design have significantly minimized the systematic problems that are frequently encountered in the conventional array design (one array per slide and ordered printing), thus further enhancing the reproducibility and accuracy of the microarray analysis results.

Recently, several additional batches of EST- or cDNA-based microarrays with different formats and elements have been reported in cotton [102, 104, 140]. Shi et al. [104] reported the fabrication of microarrays from unigene ESTs derived from 5–10 dpa ovules of the Upland cotton cv. Xuzhou 142 and using the amplicons of the EST clones as the array elements. The microarrays each consist of 11,962 uniEST elements. Using the microarrays, Shi et al. [104] comparatively studied the wild-type Xuzhou 142 versus its *fuzzless-lintless* (*fl*) mutation using the RNAs isolated from the ovules at stages of 0-, 3-, 5-, 10-, 15-, and 20-dpa. It was found that ethylene biosynthesis is one of the most significantly upregulated biochemical pathways during fiber elongation. Similarly, Wu et al. [140] also fabricated a set of microarrays from amplicons of 10,410 cDNA clones derived from −3- to 0-dpa ovules of the Upland cotton cv. DP16 (see Table 5, Wu & Dennis). The arrays were analyzed with RNAs isolated from 0-dpa whole ovules, outer integument, and inner integument/nucellus of five lintless mutation lines against the wild-type DP16. Of the 10,410 gene elements on the array, 60 to 243 were found to significantly differentially express between each pair of the wild type and mutant when the array was hybridized with the RNAs isolated from the 0-dpa whole ovules. Of these differentially expressed genes, 70.6% were upregulated and 29.4% downregulated in the fiber mutant, suggesting that the mutation caused not only gene downregulation, but also gene upregulation. However, when the whole ovule was dissected into three layers, outer integument, inner integument, and nucellus, of which cotton fibers develop from the epidermal cells of the outer integument, and analyzed with the outer integument against the inner integument and the nucellus, the number of the genes downregulated in the mutants was reduced to 13. These include an *Myb* transcription factor, a putative *homeodomain* protein, a *cyclin D* gene, and some fiber-expressed structural and metabolic genes, suggesting that these genes may be involved in the process of fiber initiation.

In summary, three batches of EST- or cDNA-based cotton microarrays were fabricated from fiber genes of either cultivated tetraploid cotton, *G. hirsutum* [104, 140], or cultivated diploid cotton, *G. arboreum* [100]. Using the microarrays, the expression of the fiber genes was profiled and comparatively analyzed at fiber initiation stage [140], elongation stage [100, 104], and secondary cell wall deposition stage [100]. However, the expression of other cotton genes such as those from nonfiber and nonovary tissues remains to profile. To fill this gap, another two batches of long oligo-based microarrays have been developed. The first batch contains approximately 21,000 gene elements per array (http://cotton.agtec.uga.edu/CottonFiber/pages/mcriarray/Array.aspx). These genes were from 52 cDNA libraries constructed from a variety of tissues and organs in a range of conditions, including drought stress and pathogen challenges, and represents tetraploid (*G. hirsutum*) and its diploid relatives (*G. arboreum* and *G. raimondii*). Of the 21,000 genes, approximately one-forth were from fiber genes and three-forth were from nonfiber and nonovary tissues (J. A. Udall, pers. communication). The second batch contains 38,716 gene elements per array. Of the gene elements, 22,409 are designed from fiber ESTs and 16,307 from nonfiber ESTs (T.A. Wilkins, pers. communication). There is no doubt that these versions of cotton microarrays will provide new tools for comprehensive functional and comparative genomics research of cottons.

### 2.5. Physical mapping

Whole-genome, BAC- and/or BIBAC-based, integrated physical/genetic maps have played a central role in genomics research of humans, plants, animals, and microbes [110, 123, 127]. This is because they provide central platforms for many areas, if not all, of modern genomics research, including large-scale transcript or gene mapping, region-targeted marker development for fine mapping and MAS of genes and QTLs, map-based gene/QTL cloning, local- and whole-genome comparative analysis, genome sequencing, and functional analysis of DNA sequences and component network. Therefore, whole-genome, BAC/BIBAC-based, integrated physical/genetic maps have been developed for a number of plant and animal species. In plants, whole-genome BAC physical maps have been developed for several species, including *Arabidopsis* [114, 118], indica rice [121], japonica rice [117], soybean [124], and maize [141]. However, whole-genome physical maps of cottons have only been initiated in several laboratories. One is the laboratory of H.-B. Zhang, Texas A&M University, College Station (Tex, USA). This laboratory is developing a whole-genome BAC/BIBAC physical map of the Upland cotton cv. TM-1 by using the latest physical mapping technology [123, 126]. The project was a collaborative effort among the laboratories of H.-B. Zhang, R. J. Kohel, USDA/ARS, College Station (Tex, USA) (who provided a part of the fund for the project), and D. M. Stelly, Texas A&M University (Tex, USA). Nearly 120,000 (∼7.3x) BIBACs and BACs selected from the TM-1 BIBAC and BAC libraries (see Table 3) have been fingerprinted and a draft BAC/BIBAC contig map has been constructed. The draft physical map consists of 5,088 contigs collectively spanning approximately 2,300 Mb of the 2,400 Mb Upland genome (unpublished). Currently, additional clones (to reach about 10x genome coverage clones) are being analyzed. Furthermore, because the Upland cotton is an allotetraploid which makes the physical map construction more complicated, several approaches are being used to sort the map contigs according to their origin of subgenomes. The laboratory of A. H. Paterson, University of Georgia (Athens, Georgia) is also working toward development of a whole-genome BAC-based physical map of the diploid species, *G. raimondii* (A. H. Paterson, pers. communication). Given the importance of physical maps for modern genome research, there is no doubt that development of a robust integrated physical/genetic map will greatly promote advanced genomics research of cottons and related species (also see below).

### 2.6. Genome sequencing

Sequence maps represent the most-fine physical maps of genomes [108]. They provide not only physical positions of and distances between genes and other components constituting a genome [142], but also their sequences and putative functions inferred from the sequences. Therefore, development of a complete genome sequence map of a species will significantly promote genomics research of the species in a variety of aspects. Because of this reason, the whole genomes of several plant and animal species have been sequenced. In plants, the genomes of two model species, *Arabidopsis* [130] and rice [132], have been completely sequenced and the genomes of several other species, including *Medicago truncatula* (http://www.medicago.org), *Lotus japonicus* (http://www.kazusa.or.jp/lotus), tomato (http://www.sgn.cornell.edu/about/tomato_sequencing.pl), maize (http://www.maizegenome.org), and soybean (http://genome.purdue.edu/isgc/Tsukuba07/ISGC_report_Apr2007.htm), are currently being sequenced.

However, there is only a limited amount of genomic sequences available for cotton and related species in GenBank. A major source of the genomic sequences of *Gossypium* species was from Hawkins et al. [143]. To understand the underlying genome size variation and evolution of *Gossypium* species, Hawkins et al. [143] constructed whole-genome shotgun libraries for *G. raimondii* (D_5_D_5_), *G. herbaceum* (A_1_A_1_), *G. exiguum* (KK), and the species that was used as the outgroup species for phylogenetic analysis of the *Gossypium* species, *Gossypioides kirkii*, with each species library containing 1920–10,368 clones. From each of the four shotgun libraries, 1,464–6,747 clones were sequenced, together covering a total length of 11.4 Mb. Annotation of these clone sequences and estimation of the copy number of each type of the sequences suggested that differential lineage-specific amplification of transposable elements is responsible for genome size variation in the *Gossypium* species. Moreover, *G. raimondii* has been selected recently by the DOE Joint Genome Institute, U.S. Department of Energy to be sequenced for genomic study of cotton and related species (http://www.jgi.doe.gov/sequencing/cspseqplans2007.html). At the first phase of the sequencing project, a whole-genome shotgun library covering about 1x of the *G. raimondii* genome will be sequenced. While this number is far from the genome coverage of clones (>6x) that is needed to assemble the sequence map of the genome, it will provide the first glimpse into the cotton genome and useful information for sequencing the entire genomes of this and other cotton key species efficiently.

## 3. APPLICATIONS OF GENOMIC TOOLS IN COTTON GENETIC IMPROVEMENT

One of the major goals of genome research is to use the genomic tools developed to promote or assist continued crop genetic improvement. In cottons, the development of the genomic resources and tools has allowed addressing many significantly scientific questions that are impossible to do so before. These include, but not limited to, construction of genome-wide genetic maps (Table 1), identification and mapping of genes and loci controlling traits underlying qualitative and quantitative inheritance (Table 2), determination of mechanisms of cotton genome evolution, and identification and determination of genes that are involved in cotton fiber initiation, elongation, and secondary cell wall biogenesis. The genomic resources and tools could be used to promote or facilitate cotton genetic improvement in numerous ways. Marker-assisted selection (MAS) is likely one of the most important and practical applications at present time and in near future. The MAS technology could offer many potential benefits to a breeding program. For instance, DNA linked to a gene of interest could be utilized in early generation of breeding cycle to improve the efficiency of selection. This approach has a particular advantage when screening for phenotypes in which the selection is expensive or difficult to perform, as is the case involving recessive or multiple genes, seasonal or geographical considerations, and late expression of the phenotype [144]. However, application of MAS in cotton breeding programs is still in its infancy as the major effort of cotton genome research in the past has been on the development of genomic resources and tools for the eventual goal of enhanced cotton genetic improvement.

**Fiber quality**

Zhang et al. [53] used a *G. anomalum* introgression line 7235 with good fiber quality properties to identify molecular markers linked to fiber-strength QTLs. A major QTL, *QTLFS1*, was detected at the Nanjing and Hainan field locations (China) and College Station, Texas, (USA). This QTL was associated with eight markers and explained more than 30% of the phenotypic variation. *QTLFS1* was first thought to be mapped to chromosome 10, however, further study showed that this QTL was located on LGD03 [67]. Guo et al. [145] showed that the specific SCAR4311920 marker could be applied to large-scale screening for the presence or absence of this major fiber strength QTL in breeding populations. The DNA markers tightly linked to this QTL could be useful for developing commercial cultivars with enhanced fiber length properties [67].

Wang et al. [76] identified a stable fiber length QTL, *qFL-D2-1*, simultaneously in four environments in Xiangzamian 2. The high degree of stability suggests this QTL might be particularly valuable for use in MAS programs. Chee et al. [59] dissected the molecular basis of genetic variation governing 15 parameters that reflect fiber length by applying a detailed RFLP map to 3,662 BC_3_F_2_ plants from 24 independently derived BC_3_ families utilizing *G. barbadense* as the donor parent. The discovery of many QTLs unique to each trait indicates that maximum genetic gain will require breeding efforts that target each trait. Lacape et al. [64] performed QTL analysis of 11 fiber properties in BC_1_, BC_2_, and BC_2_S_1_ backcross generations derived from the cross between *G. hirsutum* “Guazuncho 2” and *G. barbadense* “VH8.” They detected 15, 12, 21, and 16 QTLs for length, strength, fineness, and color, respectively, in one or more populations. The results showed that favorable alleles came from the *G. barbadense* parent for the majority of QTLs, and cases of colocalization of QTLs for different traits were more frequent than isolated positioning. Taking these QTL-rich chromosomal regions into consideration, they identified 19 regions on 15 different chromosomes as target regions for the marker-assisted introgression strategy. The availability of DNA markers linked to *G. barbadense* QTLs promises to assist breeders in transferring and maintaining valuable traits from exotic sources during cultivar development.

**Cytoplasmic male sterility**

In cotton, cytoplasmic male sterility conditioned by the D8 alloplasm (CMS-D8) is independently restored to fertility by its specific D8 restorer (D8R) and by the D2 restorer (D2R) that was developed for the D2 cytoplasmic male sterile alloplasm (CMS-D2). Zhang and Stewart [146] concluded that the two restorer loci are nonallelic, but are tightly linked with an average genetic distance of 0.93 cM. The D2 restorer gene is redesignated as *Rf1*, and *Rf2* is assigned to the D8 restorer gene. The identification of molecular markers closely linked to restorer genes of the cytoplasmic male sterile could facilitate the development of parental lines for hybrid cotton. Guo et al. [147] found that one RAPD marker fragment, designated OPV-15(300), was closely linked with the fertility-restoring gene *Rf1*. Zhang and Stewart [148] identified RAPD markers linked to the restorer gene and, furthermore, converted the three RAPD markers into reliable and genome-specific sequence tagged site (STS) markers. Liu et al. [51] determined that the *Rf1* locus is located on the long arm of chromosome 4. Two RAPD and three SSR markers were identified to be closely linked to the *Rf1* gene. These markers are restorer-specific and should be useful in MAS for developing restorer parental lines. Yin et al. [54] further constructed a high-resolution genetic map of *Rf1* containing 13 markers in a genetic distance of 0.9 cM. They constructed a physical map for the *Rf1* locus and enclosed the possible location of the *Rf1* gene to a minimum of two BAC clones spanning an interval of approximately 100 kb between two clones, designated as 081-05K and 052-01N. Work to isolate the *Rf1* gene in cotton is now in progress.

**Resistance to diseases and insect pests**

Breeding for disease resistance is of great importance in cotton breeding program. To facilitate analysis, cloning, and manipulation of the genes conferring resistance to different pathogens, including bacteria, fungi, viruses, and nematodes, He et al. [149] isolated and characterized the family of nucleotide-banding site-leucine-rich repeat (NBS-LRR)-encoding genes or resistance gene analogues (RGAs) in the Upland cotton cv. Auburn 634 genome. Genetic mapping of a sample (21 genes) of the RGAs indicated that the gene family resides on a limited number of the cotton AD-genome chromosomes with those from a single subfamily tending to cluster on the cotton genetic map and more RGAs in the A subgenome than in the D subgenome. Of the 16 RGAs mapped, two happened to be comapped with the cotton bacterial blight resistance QTLs previously mapped by Wright et al. [42]. Since nearly 80% of the genes (>40 genes) cloned to date that confer resistance to bacteria, fungi, viruses, and nematodes are contributed by the NBS-LRR gene family, the cotton RGAs of the NBS-LRR family have provided valuable tools for cloning, characterization, and manipulation of the resistant genes to different pathogens and pests in cottons.

Root-knot nematodes (RKN), *Meloidogyne incognita*, can cause severe yield loss in cotton. Wang et al. [71] identified one SSR marker CIR316 on the linkage group A03 tightly linked to a major RKN resistant gene (*rkn1*) in the resistant cultivar *G. hirsutum* “Aacla NemX.” In a companion study, a bulked segregant analysis (BSA) combined with AFLP was used to identify additional molecular markers linked to *rkn1* [72]. An AFLP marker linked to *rkn1* designated as GHACC1 was converted to a cleaved amplified polymorphic sequence (CAPS) marker. These two markers have potential for utilization in MAS. Shen et al. [68] identified RFLP markers on chromosome 7 and chromosome 11 showing significant association with RKN resistance from the Auburn 634 source, a different source of resistant germplasm than Acala NemX. The association was further confirmed by detection of a minor and a major dominant QTL on chromosomes 7 and 11, respectively, using SSR markers. Ynturi et al. [73] identified two SSR markers which together accounted for 31% of the variation in galling index. The marker BNL 3661 is mapped to the short arm of chromosome 14 while BNL 1231 to the long arm of chromosome 11. The association of two different chromosomes with RKN resistance suggests at least two genes are involved in resistance to RKN.

Bacterial blight caused by the pathogen *Xanthomonas campestris* pv. *malvacearum* (*Xcm*) is another economically important disease in cotton. Wright et al. [42] and Rungi et al. [43] both used mapped RFLP markers to investigate the chromosomal location of genes conferring resistance to the bacterial blight pathogen. The mapping data suggest that the resistance locus segregates with a marker on chromosome 14 known to be linked to the broad-spectrum *B12* resistance gene originally from African cotton cultivars. AFLPs and SSRs were also used to search for novel markers linked to the *Xcm* resistance locus to facilitate introgression of this trait into *G. barbadense* through MAS.

## 4. CONCLUDING REMARKS

A significant amount of genomic resources and tools has been available in cottons though cotton genomics research is far behind those of other major crops such as rice, maize, wheat, and soybean. These resources and tools have allowed identifying and mapping many genes and QTLs of importance to cotton fiber quality, fiber yield, and biotic and abiotic stresses and addressing several significant questions to plant biology in general and to cotton in particular. Nevertheless, many efforts are needed to further develop the resources and tools and to make the tools readily usable in applications in order to fully and effectively use them in cotton genetic improvement and biology research. In particular, the following areas of cotton genomics research should be emphasized.

(i) *Development of whole-genome BAC/BIBAC-based, integrated physical maps of cottons*. There is no whole-genome, robust BAC/BIBAC-based, integrated physical/genetic map that has been developed for cottons. The maps should be developed for at least two species of *Gossypium*. One is the Upland cotton that produces >90% of the world’s cotton whereas the other is *G. raimondii*, the species having the smallest genome among all *Gossypium* species, thus likely having highest density of genes. This research is emphasized because it has been proven in model and other species, including *Arabidopsis*, rice, *Drosophila*, human, mouse, and chicken, that whole-genome integrated physical/genetic maps provide powerful platforms and “freeways” for many, if not all, modern genetics and genomics research ([110, 123, 127]; see above). These include not only genome sequencing (see below), but also development of closely linked, user-friendly DNA markers for any region or loci of the genome, fine QTL and gene mapping (see below), map-based gene/QTL cloning, and high-throughput and high-resolution transcript (unigene EST) mapping [150]. Development of the integrated physical maps will allow rapidly and efficiently integrating all existing genetic maps, mapped genes and QTLs, and BAC and BIBAC resources and cotton unigene ESTs, and accelerate the efficiency and reduce the cost of research in all areas by manifold.

(ii) *QTL fine mapping*. Many genes and QTLs that are important to cotton fiber yield fiber quality, and biotic and abiotic stresses have been genetically mapped, but two problems are apparent. The first one is that almost all of the QTLs were mapped using F_2_, BC_1_, or early segregating generations in a single or a limited number of environments (Table 2). Since quantitative traits are readily subjected to environmental variation, the mapping results using the early generations in a single or a limited number of environments would vary from experiments to experiments [59, 60, 106]. The other problem is that the genetic distances between DNA markers and most of the QTLs are too far to be used for MAS. Therefore, it is of significance to fine map the QTLs using large and advanced generation or homozygous populations, such as RILs and DHs, in multiple environments, and closely linked DNA markers, for which advantage of integrated physical maps could be taken. In addition to accurate mapping of the QTLs and development of DNA markers that are well-suited (closely linked and user-friendly) for MAS, fine mapping is also an essential step toward the final isolation of the QTL genes by map-based cloning [134]. The isolated genes are not only the sources for molecular breeding via genetic transformation, but also the most desirable for marker development for MAS because there is no recombination between the gene and its derived marker.

(iii) *Sequencing of one or more key cotton genomes*. While it is costly using the current sequencing technology, whole-genome sequencing is a most-efficient method to discover and decode all cotton genes and provides a most-desired and most-fine integrated physical and genetic map of the cotton genome. Comparative genomics studies demonstrated that the gene contents and orders are highly conserved among the genomes of *Gossypium* species even they are significantly different in genome size [16, 24]. Based on this result, *G. raimondii* is an excellent choice to be sequenced because it has the smallest genome among all *Gossypium* species though it is not cultivated. If an integrated physical map is available for the major cultivated cotton, *G. hirsutum*, that has a three-fold larger genome than *G. raimondii*, the sequence information of *G. raimondii* could be readily transferred to the cultivated cotton by using the BAC end sequences of its integrated physical map as anchors.

(iv) *ESTs from nonfiber and nonovary tissues and fibers at the secondary cell wall deposition stage*. As shown above, the number of cotton ESTs available in GenBank has been increased significantly in the past few years; however, the distribution of the ESTs among tissue sources are extremely biased. The numbers of ESTs from both nonfiber/nonovary tissues and fibers at the secondary cell wall deposition stage (15–45 dpa), particularly after 20 dpa, are especially small. The former set of expressed genes, despite of not directly contributing to fiber yield and quality, is of significance to fiber yield and quality, whereas there is no doubt that the later set of expressed genes directly contribute to the fiber strength.

(v) *Profiling and identification of genes involved in individual biological processes and conditions with emphasis on those involved in fiber development*. Development and availability of cDNA- or unigene EST-based microarrays have provided unprecedented opportunities for research of molecular biology, functional genomics, and evolutionary genomics, however, cotton research in these regards are very limited. Identifying and characterizing genes that are involved in the processes of fiber development, plant growth and development, and biotic and abiotic stresses will greatly facilitate our understanding of underlying molecular basis of these processes in cottons, and thus, enhance breeders’ ability to cotton genetic improvement.

(vi) *Translating the gene activities or expressions at different tissues and stages into fiber yield and fiber quality, thus assisting in cotton breeding.* The genes that are involved in fiber initiation [104, 140], elongation [100, 104], and secondary cell wall deposition [100] have been identified from several genotypes of cottons, but it is unknown about what the up- or downregulation, or active expression of fiber genes at a developmental stage and organ means to final fiber yield and/or quality. For instance, does the active expression of a gene at fiber elongation stage in fiber suggest longer fibers? Studies in this regard are essential to use the gene expression data in cotton germplasm analysis and breeding.

## Figures and Tables

**Table 1:** Genetic maps constructed for *Gossypium* species.

Marker type	Total loci	Map distance	Population^(a)^	Cross type^(b)^
AFLP	176	773 cM	F2	GN × GAU	[26]
AFLP	213	931 cM	F2	GN × GAU	[26]
AFLP, SSR, and RFLP	392	3,287 cM	F2	GH × GB	[27]
AFLP, SSR, and RFLP	888	4,400 cM	BC1	GH × GB	[28]
AFLP, SSR, and RFLP	1,160	5,519 cM	BC1	GH × GB	[29]
RFLP	275	1,147 cM	F2	GAR × GHE	[16]
RFLP	284	1,503 cM	F2 and F3	GH × GH	[30]
RFLP	589	4,259 cM	F2	GH × GTO	[31]
RFLP	705	4,675 cM	F2	GH × GB	[23]
RFLP	763	1,493 cM	F2	GT × GR	[24]
RFLP	2,584	4,448 cM	F2	GH × GB	[24]
RFLP and RAPD	355	4,766 cM	F2	GH × GB	[25]
SRAP	237	3,031 cM	F2	GH × GB	[32]
SRAP, SSR, and RAPD	566	5,142 cM	F2	GH × GB	[33]
SRAP, SSR, RAPD and REMAP	1,029	5,472 cM	F2	GH × GB	[34]
SSR	193	1,277 cM	RIL	GH × GB	[35]
SSR	433	2,126 cM	RIL	GH × GB	[36]
SSR	442	4,331 cM	BC1	GH × GB	[37]
SSR	444	3,263 cM	DH	GH × GB	[37]
SSR	624	5,644 cM	BC1	GH × GB	[38]
SSR	907	5,060 cM	BC1	GH × GB	[39]
SSR	1,790	3,426 cM	BC1	GH × GB	[40]
SSR and RAPD	489	3,315 cM	DH	GH × GB	[41]

^(a)^RIL = recombinant inbred line, and DH = doubled haploid.

^(b)^GH = *G. hirsutum*, GB = *G. barbadense*, GTO = *G. tomentosum*, GR = *G. raimondii*, GAR = *G. arboretum*, GHE = *G. herbaceum*, GN = *G. nelsonii*, and GAU = *G. australe*.

**Table 2:** QTLs identified for various traits in cottons.

Traits/genes	Parental materials	Reference
Resistance to the bacterial blight pathogen	Empire B2/B3/B2b6, S295 and Pima S-7*	[42]
Resistance to the bacterial blight pathogen	CS50 and Pima S-7*	[43]
Density of leaf and stem trichomes	Pima S-7 and Empire B2b6*	[44]
Fiber quality and yield	CAMD-E and Sea Island Seaberry*	[45]
Agronomic and fiber traits	MARCABUCAG8US-1-88 and HS46	[46]
Cotton leaf morphology and other traits	Seaberry and Deltapine 61 with morphological mutants*	[47]
Productivity and quality	Siv’on and F-177*	[48]
Physiological variables and crop productivity	Siv’on and F-177*	[49]
Fiber quality	TM-1 and 3-79*	[25]
Yield components, fiber, flowering date et al.	TM-1 and 3-79*	[50]
*Rf1* fertility-restoring gene	CMS and the restoring lines	[51]
Fiber quality	Siv’on and F-177*	[52]
Fiber strength	7235 and TM-1	[53]
*Rf1* fertility-restoring gene	XiangyuanA, ZMS12A, Sumian16A and 0-613-2R	[54]
Fiber-related traits	Acala-44 and Pima S-7*	[27]
Agronomic and fiber quality traits	MD5678ne and Prema	[55]
Fiber and yield traits	MARCABUCAG8US-1-88, HS46, MD5678ne et al.	[56]
Resistance to Verticillium wilt	Pima S-7 and Acala 44*	[57]
Fiber elongation	Tamcot 2111 and Pima S6*	[58]
Fiber length, length uniformity, and short fiber content	Tamcot 2111 and Pima S6*	[59]
Fiber fineness and micronaire (MIC)	Tamcot 2111 and Pima S6*	[60]
Li1, Li2, N1, Fbl, n2, sma-4(ha), and sma-4(fz)	Pima S-7, Li1, Li2, N1, Fbl,n2, SMA4, A1-97*	[61]
Leaf morphology	TMS-22 and WT936*	[31]
Leaf morphological traits and chlorophyll content	TM-1 and Hai 7124	[62]
Fiber quality traits	TM1 and Pima 3-79*	[35]
Leaf and stem pubescence	Guazuncho 2 and VH8-4602*	[63]
Fiber quality	Guazuncho 2 and VH8*	[64]
Lint percentage and fiber quality traits	Yumian 1 and T586	[65]
Fiber traits	Handan208 and Pima90*	[33]
Fiber yield and yield components	Handan 208 and Pima 90*	[66]
Fiber quality and yield component	Handan 208 and Pima 90*	[34]
Fiber traits	7235, TM-1, HS427-10, PD6992 and SM3	[67]
Root-knot nematode resistance gene	M-120 RNR and Pima S-6*	[68]
Fiber and yield component traits	7235 and TM-1	[69]
Fiber quality and yield components	7235 and TM-1	[70]
Root-knot nematode resistance gene *(rkn1)*	Acala SJ-2, Acala NemX, and Pima S-7*	[71]
Root-knot nematode resistance gene *(rkn1)*	Acala SJ-2 and Acala NemX	[72]
Root-knot nematode resistance gene	Resistant near isoline and susceptible near isoline	[73]
Lint percentage and morphological marker genes	TM-1 and T586	[74]
Fiber-related traits	TM-1 and 3-79*	[36]
Yield, yield component and fiber quality	Near-isogenicBC5S1 chromosome substitution lines, TM-1	[75]
Plant architecture traits	Zhongmiansuo12 and 8891	[76]
Fiber quality traits	Zhongmiansuo12 and 8891	[77]
Yield and yield-component traits	Zhongmiansuo12 and 8891	[78]

*Interspecific cross.

**Table 3:** Upland cotton BAC and BIBAC libraries that have been published or are accessible to the public (as of May 2007).

Genotype	Mean insert size (kb)	No. of clones	Genome equivalents	Vector^(a)^	Cloning site	References/locations where libraries are available
Tamcot HQ95	93	51,353	2.3x	pBeloBAC11	*Hin*dIII	http://hbz7.tamu.edu
Auburn 623	140	44,160	2.7x	pBeloBAC11	BamHI	http://hbz7.tamu.edu
Texas Marker-1	130	76,800	4.4x	pCLD04541	*Bam*HI	http://hbz7.tamu.edu
Texas Marker-1	175	76,800	6.0x	pECBAC1	*Eco*RI	http://hbz7.tamu.edu
Maxxa	137	129,024	8.3x	pCUGI-1	*Hin*dIII	[79]
0-613-2R	130	97,825	5.7x	pIndigoBAC-5	*Hin*dIII	[80]

^(a)^The vectors, pBeloBAC11 (Kim et al. [82]), pECBAC1 (Frijters et al. [82]), pCUGI-1 [79], and pIndigoBAC-5 (http://www.epibio.com/item.asp?ID=328), are BAC vectors whereas pCLD04541 is plant-transformation-competent BIBAC vector (http://www.jic.bbsrc.ac.uk/staff/ian-bancroft/vectorspage.htm; [83]) that can be directly transformed into cotton plants via *Agrobacterium*.

**Table 4:** Summary of ESTs of major crops available in GenBank (as of April 27, 2007).

Species	Genome^(a)^	No. of ESTs
Cotton and related species (*Gossypium* species):		
*G. hirsutum* (Upland cotton)	(AADD)_1_	177,154
*G. raimondii*	D_5_D_5_	63,577
*G. arboreum*	A_2_A_2_	39,232
*G. barbadense* (Sea Island)	(AADD)_2_	1,023
*G. herbaceum* var. africanum	A_1_A_1_	247

Total:		**281,233**

Rice and Related Species (*Oryza* species):		
*O. sativa* (rice)	AA	1,211,447
*O. minuta*	BBCC	5,760
*O. grandiglumis*	CCDD	128

Total:		**1,217,335**

Maize and Related Species (*Zea* species):		
*Z. mays* (maize)		1,161,241

Total:		**1,161,241**

Wheat and Related Species (*Triticum* and *Aegilops* species):		
*T. aestivum* (wheat)	AABBDD	1,050,131
*T. monococcum*	AA	10,139
*T. turgidum* ssp. *durum*	AABB	8,924
*T. turgidum*	AABB	1,938
*Ae. speltoides*	BB	4,315
*Ae. tauschii*	DD	116

Total:		**1,075,563**

Soybean and Related Species (*Glycine* species):		
*G. max* (soybean)	GG	371,817
*G. soja*	GG	18,511
*G. clandestina*	A_1_A_1_	931

Total:		**391,259**

^(a)^There is no relationship in the genome letter designation between genera, but there is relationship in the genome letter designation between species within a genus, the species with the same genome letter being closely related.

**Table 5:** Summary of cotton ESTs (as of May 2007).

Genotypes	Library name	Tissues used	No. of ESTs	No. of unigenes	Authors	References
*Gossypium arboreum* (A_2_):					
AKA8401	GA-Ea	7- to 10-dpa fibers	46,603		Wing et al.
		(normalized)			Arpat et al.
	Subtotal		**46,603**	**13,947**		[100]

*G. Raimondii* (D_5_):							
GN34	GR_Ea	Whole seedlings	33,671		Udall et al.		
		with 1st true leaves				
GN34	GR-Eb	−3-dpa buds to	33,061		Udall et al.	
		+3-dpa bolls				
	Subtotal		**68,732**			

*G. hirsutum* (AD_1_):						
Coker 312	GH_MD	8- to 10-dpa boll	1,144		Allen
		(irrigated)			
Coker 312	GH_MDDS	8- to 10-dpa boll	1,238		Allen
		(drought stressed)			
Coker 312	GH_LDI	15- to 20-dpa boll	1,799		Allen & Payton
		(irrigated)			
Coker 312	GH_LDDI	15- to 20-dpa boll	1,409		Allen & Payton
		(drought stressed)			
Acala Maxxa	GH_BNL	5-dpa fibers	8,022		Blewitt & Burr	[101]
Xu-142	GH_FOX	0- to 5-dpa ovule and	7,997		Gou & Chen	
		1- to 22-dpa fibers				
Deltapine 90	GH_SCW	2nd versus 1st primary	7,385		Haigler &
		fibers			Wilkerson
Zhongmian 12	GH_SUO	0-dpa ovules	1,240		Suo & Xue
Deltapine 16	GH-CHX	−3- to 0-dpa ovules	7,631		Wu & Dennis
Deltapine 16	GH_OCF	0-dpa ovules	867		Wu and Dennis
Deltapine 16	GH_ON	0-dpa ovules	5,903		Wu & Dennis
		(normalized)			
Stv 7A gl	GH_ECT	18 h etiolated seedlings	2,880		Chapman	
Delta emerald	GH_CRH	Root and hypocotyls	1,464		Dowd & McFadden	
Delta emerald	GH_CFUS	RH tissues infected with	820		Dowd &	
		*Fusarium oxysporum*			McFadden	
Sicot	GH_LSL	S9i leaves, late season	1,810		Faivre-Nitschke	
					& Dennis	
Coker 312	GH_SDL	Seedlings (control)	1,918		Klueva et al.	
Coker 312	GH_SDLD	Seedlings (drought	1,142		Klueva & Nguyen	
		stressed)				
Coker 312	GH_SDCH	Seedling (chilling	576		Klueva & Nguyen	
		stressed)				
Deltapine 16	GH_IME	Immature embryo	1,536		Liu & Dennis	
Im216	GH_IMX	Leaf 8, 14, 20, 30, 45,	1,134		Patil et al.	
		60 hpi *Xanthomonas*				
AcB4Blnb7	GH_ACXE	Leaf 8 + 14 hpi	647		Phillips et al.	
		*Xanthomonas*				
AcB4Blnb7	GH_ACXM	Leaf 20 + 30 hpi	1,328		Phillips et al.	
		*Xanthomonas*				
AcB4Blnb7	GH_ACXL	Leaf 45 + 60 hpi	862		Phillips et al.	
		*Xanthomonas*				
T25	GH_pAR	Leaves	1,230		Trolinder	
DES119	GH_STEM	Mature stem	8,643		Taliercio	
DP62	GH_ECOT	Etiolated cotyledon	2,772		Ni & Trelease	
91-D-92	GH_CBAZ	Ball abscission zone	1,306		Wan & Wing	
			185,198^(a)^	51,107		[102]
Texas Marker-1	GH_TMO	−3- to 3-dpa ovules	32,789	8,540	Chen	[103]
−1 (TM-1)	Not available	5- to 10-dpa fibers	29,992	12,992	Zhu	[104]
Xuzhou 142						
(Xu-142)						
	Subtotal		**132,644**			

	Total		**247,979**			

^(a)^The number was the sum of numbers of all ESTs above the line including those of *G. arboreum*, *G. raimondii* and *G. hirsutm* [102]. Of the 247,979 cotton ESTs, 187,014 (75.4%) were from developing fibers or ovules whereas 160,965 (24.6%) from nonfiber or nonovule organs.

## References

[1] Endrizzi JE, Turcotte EL, Kohel RJ, Kohel RJ, Lewis CF (1984). Qualitative genetics, cytology, and cytogenetics. *Cotton*.

[2] Wendel JF, Cronn RC (2003). Polyploidy and the evolutionary history of cotton. *Advances in Agronomy*.

[3] Hendrix B, Stewart JMcD (2005). Estimation of the nuclear DNA content of *Gossypium species*. *Annals of Botany*.

[4] Endrizzi JE, Turcotte EL, Kohel RJ (1985). Genetics, cytology, and evolution of *Gossypium*. *Advances in Genetics*.

[5] Percival AE (1987). The national collection of *Gossypium germplasm*.

[6] Bennett MD, Leitch IJ (1995). Nuclear DNA amounts in angiosperms. *Annals of Botany*.

[7] Leitch IJ, Bennett MD (1997). Polyploidy in angiosperms. *Trends in Plant Science*.

[8] Masterson J (1994). Stomatal size in fossil plants: evidence for polyploid in majority of angiosperms. *Science*.

[9] Lundin LG (1993). Evolution of the vertebrate genome as reflected in paralogous chromosomal regions in man and the house mouse. *Genomics*.

[10] Postlethwait JH, Yan Y-L, Gates MA (1998). Vertebrate genome evolution and the zebrafish gene map. *Nature Genetics*.

[11] Sidow A (1996). Gen(om)e duplications in the evolution of early vertebrates. *Current Opinion in Genetics & Development*.

[12] Spring J (1997). Vertebrate evolution by interspecific hybridisation—are we polyploid?. *FEBS Letters*.

[13] Wendel JF, Schnabel A, Seelanan T (1995). Bidirectional interlocus concerted evolution following allopolyploid speciation in cotton (*Gossypium*). *Proceedings of the National Academy of Sciences of the United States of America*.

[14] Wendel JF (1989). New world tetraploid cottons contain old world cytoplasm. *Proceedings of the National Academy of Sciences of the United States of America*.

[15] Small RL, Ryburn JA, Cronn RC, Seelanan T, Wendel JF (1998). The tortoise and the hare: choosing between noncoding plastome and nuclear A*dh* sequences for phylogeny reconstruction in a recently diverged plant group. *American Journal of Botany*.

[16] Desai A, Chee PW, Rong J, May OL, Paterson AH (2006). Chromosome structural changes in diploid and tetraploid a genomes of *Gossypium*. *Genome*.

[17] Small RL, Ryburn JA, Wendel JF (1999). Low levels of nucleotide diversity at homoeologous A*dh* loci in allotetraploid cotton (*Gossypium* L.). *Molecular Biology and Evolution*.

[18] Seelanan T, Schnabel A, Wendel JF (1997). Congruence and consensus in the cotton tribe (Malvaceae). *Systematic Botany*.

[19] Wendel JF, Albert VA (1992). Phylogenetics of the cotton genus (*Gossypium* L.): character-state weighted parsimony analysis of chloroplast DNA restriction site data and its systematic and biogeographic implications. *Systematic Botany*.

[20] Beasley JO (1942). Meiotic chromosome behavior in species hybrids, haploids, and induced polyploids of *Gossypium*. *Genetics*.

[21] Stelly DM (1993). Interfacing cytogenetics with the cotton genome mapping effort.

[22] Kim HJ, Triplett BA (2001). Cotton fiber growth in planta and in vitro. Models for plant cell elongation and cell wall biogenesis. *Plant Physiology*.

[23] Reinisch AJ, Dong J-M, Brubaker CL, Stelly DM, Wendel JF, Paterson AH (1994). A detailed RFLP map of cotton, *Gossypium hirsutum x Gossypium barbadense*: chromosome organization and evolution in a disomic polyploid genome. *Genetics*.

[24] Rong JK, Abbey C, Bowers JE (2004). A 3347-locus genetic recombination map of sequence-tagged sites reveals types of genome organization, transmission and evolution of cotton (*Gossypium*). *Genetics*.

[25] Kohel RJ, Yu J, Park Y-H, Lazo GR (2001). Molecular mapping and characterization of traits controlling fiber quality in cotton. *Euphytica*.

[26] Brubaker CL, Brown AHD (2003). The use of multiple alien chromosome addition aneuploids facilitates genetic linkage mapping of the *Gossypium G* genome. *Genome*.

[27] Mei M, Syed NH, Gao W (2004). Genetic mapping and QTL analysis of fiber-related traits in cotton (*Gossypium*). *Theoretical and Applied Genetics*.

[28] Lacape J-M, Nguyen T-B, Thibivilliers S (2003). A combined RFLP-SSR-AFLP map of tetraploid cotton based on a *Gossypium hirsutum x Gossypium barbadense* backcross population. *Genome*.

[29] Nguyen T-B, Giband M, Brottier P, Risterucci A-M, Lacape J-M (2004). Wide coverage of the tetraploid cotton genome using newly developed microsatellite markers. *Theoretical and Applied Genetics*.

[30] Ulloa M, Meredith WR, Shappley ZW, Kahler AL (2002). RFLP genetic linkage maps from four F2.3 populations and a joinmap of *Gossypium hirsutum* L.. *Theoretical and Applied Genetics*.

[31] Waghmare VN, Rong J, Rogers CJ, Pierce GJ, Wendel JF, Paterson AH (2005). Genetic mapping of a cross between *Gossypium hirsutum* (cotton) and the Hawaiian endemic, *Gossypium tomentosum*. *Theoretical and Applied Genetics*.

[32] Lin Z, Zhang X, Nie Y, He D, Wu M (2003). Construction of a genetic linkage map for cotton based on SRAP. *Chinese Science Bulletin*.

[33] Lin Z, He D, Zhang X (2005). Linkage map construction and mapping QTL for cotton fibre quality using SRAP, SSR and RAPD. *Plant Breeding*.

[34] He D-H, Lin Z-X, Zhang X-L (2007). QTL mapping for economic traits based on a dense genetic map of cotton with PCR-based markers using the interspecific cross of *Gossypium hirsutum* x *Gossypium barbadense*. *Euphytica*.

[35] Park Y-H, Alabady MS, Ulloa M (2005). Genetic mapping of new cotton fiber loci using EST-derived microsatellites in an interspecific recombinant inbred line cotton population. *Molecular Genetics and Genomics*.

[36] Frelichowski Jr JE, Palmer MB, Main D (2006). Cotton genome mapping with new microsatellites from Acala ‘Maxxa’ BAC-ends. *Molecular Genetics and Genomics*.

[37] Song X, Wang K, Guo W, Zhang J, Zhang T (2005). A comparison of genetic maps constructed from haploid and BC1 mapping populations from the same crossing between *Gossypium hirsutum* L. and *Gossypium barbadense* L.. *Genome*.

[38] Han Z-G, Guo W-Z, Song X-L, Zhang T-Z (2004). Genetic mapping of EST-derived microsatellites from the diploid *Gossypium arboreum* in allotetraploid cotton. *Molecular Genetics and Genomics*.

[39] Han Z, Wang C, Song X (2006). Characteristics, development and mapping of *Gossypium hirsutum* derived EST-SSRs in allotetraploid cotton. *Theoretical and Applied Genetics*.

[40] Guo W, Cai P, Wang C (2007). A microsatellite-based, gene-rich linkage map reveals genome structure, function and evolution in *Gossypium*. *Genetics*.

[41] Zhang J, Guo W, Zhang T (2002). Molecular linkage map of allotetraploid cotton (*Gossypium hirsutum* L. x *Gossypium barbadense* L.) with a haploid population. *Theoretical and Applied Genetics*.

[42] Wright RJ, Thaxton PM, El-Zik KM, Paterson AH (1998). D-subgenome bias of *Xcm* resistance genes in tetraploid *Gossypium* (cotton) suggests that polyploid formation has created novel avenues for evolution. *Genetics*.

[43] Rungis D, Llewellyn D, Dennis ES, Lyon BR (2002). Investigation of the chromosomal location of the bacterial blight resistance gene present in an Australian cotton (*Gossypium hirsutum* L.) cultivar. *Australian Journal of Agricultural Research*.

[44] Wright RJ, Thaxton PM, El-Zik KM, Paterson AH (1999). Molecular mapping of genes affecting pubescence of cotton. *Journal of Heredity*.

[45] Jiang C-X, Wright RJ, El-Zik KM, Paterson AH (1998). Polyploid formation created unique avenues for response to selection in *Gossypium* (cotton). *Proceedings of the National Academy of Sciences of the United States of America*.

[46] Shappley ZW, Jenkins JN, Zhu J, McCarty JC (1998). Quantitative trait loci associated with agronomic and fiber traits of upland cotton. *Journal of Cotton Science*.

[47] Jiang C, Wright RJ, Woo SS, DelMonte TA, Paterson AH (2000). QTL analysis of leaf morphology in tetraploid *Gossypium* (cotton). *Theoretical and Applied Genetics*.

[48] Saranga Y, Menz M, Jiang C-X, Wright RJ, Yakir D, Paterson AH (2001). Genomic dissection of genotype x environment interactions conferring adaptation of cotton to arid conditions. *Genome Research*.

[49] Saranga Y, Jiang C-X, Wright RJ, Yakir D, Paterson AH (2004). Genetic dissection of cotton physiological responses to arid conditions and their inter-relationships with productivity. *Plant, Cell and Environment*.

[50] Ren L-H, Guo W-Z, Zhang T-Z (2002). Identification of quantitative trait loci (QTLs) affecting yield and fiber properties in chromosome 16 in cotton using substitution line. *Acta Botanica Sinica*.

[51] Liu L, Guo W, Zhu X, Zhang T (2003). Inheritance and fine mapping of fertility restoration for cytoplasmic male sterility in *Gossypium hirsutum* L.. *Theoretical and Applied Genetics*.

[52] Paterson AH, Saranga Y, Menz M, Jiang C-X, Wright RJ (2003). QTL analysis of genotype x environment interactions affecting cotton fiber quality. *Theoretical and Applied Genetics*.

[53] Zhang T, Yuan Y, Yu J, Guo W, Kohel RJ (2003). Molecular tagging of a major QTL for fiber strength in Upland cotton and its marker-assisted selection. *Theoretical and Applied Genetics*.

[54] Yin J, Guo W, Yang L, Liu L, Zhang T (2006). Physical mapping of the Rf1 fertility-restoring gene to a 100 kb region in cotton. *Theoretical and Applied Genetics*.

[55] Ulloa M, Meredith Jr WR (2000). Genetic linkage map and QTL analysis of agronomic and fiber traits in a intraspecific population. *Journal of Cotton Science*.

[56] Ulloa M, Saha S, Jenkins JN, Meredith WR, McCarty JC, Stelly DM (2005). Chromosomal assignment of RFLP linkage groups harboring important QTLs on an intraspecific cotton (*Gossypium hirsutum* L.) joinmap. *Journal of Heredity*.

[57] Bolek Y, El-Zik KM, Pepper AE (2005). Mapping of verticillium wilt resistance genes in cotton. *Plant Science*.

[58] Chee P, Draye X, Jiang CX (2005). Molecular dissection of interspecific variation between *Gossypium hirsutum* and *Gossypium barbadense* (cotton) by a backcross-self approach: I. Fiber elongation. *Theoretical and Applied Genetics*.

[59] Chee P, Draye X, Jiang CX (2005). Molecular dissection of interspecific variation between *Gossypium hirsutum* and *Gossypium barbadense* (cotton) by a backcross-self approach: III. Fiber length. *Theoretical and Applied Genetics*.

[60] Draye X, Chee P, Jiang C-X (2005). Molecular dissection of interspecific variation between *Gossypium hirsutum* and *G. barbadense* (cotton) by a backcross-self approach: II. Fiber fineness. *Theoretical and Applied Genetics*.

[61] Rong J, Pierce GJ, Waghmare VN (2005). Genetic mapping and comparative analysis of seven mutants related to seed fiber development in cotton. *Theoretical and Applied Genetics*.

[62] Song X-L, Guo W-Z, Han Z-G, Zhang T-Z (2005). Quantitative trait loci mapping of leaf morphological traits and chlorophyll content in cultivated tetraploid cotton. *Journal of Integrative Plant Biology*.

[63] Lacape J-M, Nguyen TB (2005). Mapping quantitative trait loci associated with leaf and stem pubescence in cotton. *Journal of Heredity*.

[64] Lacape J-M, Nguyen T-B, Courtois B (2005). QTL analysis of cotton fiber quality using multiple *Gossypium hirsutum x Gossypium barbadense* backcross generations. *Crop Science*.

[65] Zhang Z-S, Xiao Y-H, Luo M (2005). Construction of a genetic linkage map and QTL analysis of fiber-related traits in upland cotton (*Gossypium hirsutum* L.). *Euphytica*.

[66] He D-H, Lin Z-X, Zhang X-L (2005). Mapping QTLs of traits contributing to yield and analysis of genetic effects in tetraploid cotton. *Euphytica*.

[67] Shen X, Guo W, Zhu X (2005). Molecular mapping of QTLs for fiber qualities in three diverse lines in Upland cotton using SSR markers. *Molecular Breeding*.

[68] Shen X, Becelaere GV, Kumar P, Davis RF, May OL, Chee P (2006). QTL mapping for resistance to root-knot nematodes in the M-120 RNR Upland cotton line (*Gossypium hirsutum* L.) of the Auburn 623 RNR source. *Theoretical and Applied Genetics*.

[69] Shen X, Zhang T, Guo W, Zhu X, Zhang X (2006). Mapping fiber and yield QTLs with main, epistatic, and QTL x environment interaction effects in recombinant inbred lines of Upland cotton. *Crop Science*.

[70] Shen X, Guo W, Lu Q, Zhu X, Yuan Y, Zhang T (2007). Genetic mapping of quantitative trait loci for fiber quality and yield trait by RIL approach in Upland cotton. *Euphytica*.

[71] Wang C, Ulloa M, Roberts PA (2006). Identification and mapping of microsatellite markers linked to a root-knot nematode resistance gene (*rkn1*) in Acala NemX cotton (*Gossypium hirsutum* L.). *Theoretical and Applied Genetics*.

[72] Wang C, Roberts PA (2006). Development of AFLP and derived CAPS markers for root-knot nematode resistance in cotton. *Euphytica*.

[73] Ynturi P, Jenkins JN, McCarty JC, Gutierrez OA, Saha S (2006). Association of root-knot nematode resistance genes with simple sequence repeat markers on two chromosomes in cotton. *Crop Science*.

[74] Guo W-Z, Ma G-J, Zhu Y-C, Yi C-X, Zhang T-Z (2006). Molecular tagging and mapping of quantitative trait loci for lint percentage and morphological marker genes in upland cotton. *Journal of Integrative Plant Biology*.

[75] Saha S, Jenkins JN, Wu J (2006). Effects of chromosome-specific introgression in upland cotton on fiber and agronomic traits. *Genetics*.

[76] Wang B-H, Wu Y-T, Huang N-T, Zhu X-F, Guo W-Z, Zhang T-Z (2006). QTL mapping for plant architecture traits in upland cotton using RILs and SSR markers. *Acta Genetica Sinica*.

[77] Wang B, Guo W, Zhu X, Wu Y, Huang N, Zhang T (2006). QTL mapping of fiber quality in an elite hybrid derived-RIL population of upland cotton. *Euphytica*.

[78] Wang B, Guo W, Zhu X, Wu Y, Huang N, Zhang T (2007). QTL mapping of yield and yield components for elite hybrid derived-RILs in Upland cotton. *Journal of Genetics and Genomics*.

[79] Tomkins JP, Peterson DG, Yang TJ (2001). Development of genomic resources for cotton (*Gossypium hirsutum* L.): BAC library construction, preliminary STC analysis, and identification of clones associated with fiber development. *Molecular Breeding*.

[80] Yin J-M, Guo W-Z, Zhang T-Z (2006). Construction and identification of bacterial artificial chromosome library for 0-613-2R in upland cotton. *Journal of Integrative Plant Biology*.

[81] Kim U-J, Birren BW, Slepak T (1996). Construction and Characterization of a Human Bacterial Artificial Chromosome Library. *Genomics*.

[82] Frijters ACG, Zhang Z, van Damme M (1997). Construction of a bacterial artificial chromosome library containing large Eco RI and Hin dIII genomic fragments of lettuce. *Theoretical and Applied Genetics*.

[83] Tao Q, Zhang H-B (1998). Cloning and stable maintenance of DNA fragments over 300 kb in Escherichia coli with conventional plasmid-based vectors. *Nucleic Acids Research*.

[84] Saghai Maroof MA, Biyashev RM, Yang GP, Zhang Q, Allard RW (1994). Extraordinarily polymorphic microsatellite DNA in barley: species diversity, chromosomal locations, and population dynamics. *Proceedings of the National Academy of Sciences of the United States of America*.

[85] Reddy OUK, Pepper AE, Abdurakhmonov I (2001). New dinucleotide and trinucleotide microsatellite marker resources for cotton genome research. *Journal of Cotton Science*.

[86] Liu S, Saha S, Stelly D, Burr B, Cantrell RG (2000). Chromosomal assignment of microsatellite loci in cotton. *Journal of Heredity*.

[87] Lacape J-M, Dessauw D, Rajab M, Noyer J-L, Hau B (2007). Microsatellite diversity in tetraploid *Gossypium germplasm*: assembling a highly informative genotyping set of cotton SSRs. *Molecular Breeding*.

[88] Liu DQ, Guo XP, Lin ZX, Nie YC, Zhang XL (2006). Genetic diversity of Asian cotton (*Gossypium arboreum* L.) in China evaluated by microsatellite analysis. *Genetic Resources and Crop Evolution*.

[89] Rungis D, Llewellyn D, Dennis ES, Lyon BR (2005). Simple sequence repeat (SSR) markers reveal low levels of polymorphism between cotton (*Gossypium hirsutum* L.) cultivars. *Australian Journal of Agricultural Research*.

[90] Zhang J, Lu Y, Cantrell RG, Hughs E (2005). Molecular marker diversity and field performance in commercial cotton cultivars evaluated in the southwestern USA. *Crop Science*.

[91] Connell JP, Pammi S, Iqbal MJ, Huizinga T, Reddy AS (1998). A high through-put procedure for capturing microsatellites from complex plant genomes. *Plant Molecular Biology Reporter*.

[92] Qureshi SN, Saha S, Kantety RV, Jenkins JN (2004). Molecular biology and physiology: EST-SSR: a new class of genetic markers in cotton. *Journal of Cotton Science*.

[93] Lichtenzveig J, Scheuring C, Dodge J, Abbo S, Zhang H-B (2005). Construction of BAC and BIBAC libraries and their applications for generation of SSR markers for genome analysis of chickpea, *Cicer arietinum* L. *Theoretical and Applied Genetics*.

[94] Blenda A, Scheffler J, Scheffler B (2006). CMD: a cotton microsatellite database resource for *Gossypium* genomics. *BMC Genomics*.

[95] Chee PW, Rong J, Williams-Coplin D, Schulze SR, Paterson AH (2004). EST derived PCR-based markers for functional gene homologues in cotton. *Genome*.

[96] Saha S, Karaca M, Jenkins JN, Zipf AE, Reddy OUK, Kantety RV (2003). Simple sequence repeats as useful resources to study transcribed genes of cotton. *Euphytica*.

[97] Taliercio E, Allen RD, Essenberg M (2006). Analysis of ESTs from multiple *Gossypium hirsutum* tissues and identification of SSRs. *Genome*.

[98] Guo W, Wang W, Zhou B, Zhang T (2006). Cross-species transferability of *G. arboreum*-derived EST-SSRs in the diploid species of *Gossypium*. *Theoretical and Applied Genetics*.

[99] Wang K, Song X, Han Z (2006). Complete assignment of the chromosomes of *Gossypium hirsutum* L. by translocation and fluorescence in situ hybridization mapping. *Theoretical and Applied Genetics*.

[100] Arpat A, Waugh M, Sullivan JP (2004). Functional genomics of cell elongation in developing cotton fibers. *Plant Molecular Biology*.

[101] Haigler CH, Zhang D, Wilkerson CG (2005). Biotechnological improvement of cotton fibre maturity. *Physiologia Plantarum*.

[102] Udall JA, Swanson JM, Haller K (2006). A global assembly of cotton ESTs. *Genome Research*.

[103] Yang SS, Cheung F, Lee JJ (2006). Accumulation of genome-specific transcripts, transcription factors and phytohormonal regulators during early stages of fiber cell development in allotetraploid cotton. *The Plant Journal*.

[104] Shi Y-H, Zhu S-W, Mao X-Z (2006). Transcriptome profiling, molecular biological, and physiological studies reveal a major role for ethylene in cotton fiber cell elongation. *The Plant Cell*.

[105] Ulloa M, Brubaker C, Chee P, Kole C (2006). Cotton. *Genome Mapping & Molecular Breeding*.

[106] Rong J, Feltus FA, Waghmare VN (2007). Meta-analysis of polyploid cotton QTL shows unequal contributions of subgenomes to a complex network of genes and gene clusters implicated in lint fiber development. *Genetics*.

[107] He L, Du C, Li Y, Scheuring C, Zhang H-B, Liu Z (2007). Large-insert bacterial clone libraries and their applications. *Aquaculture Genome Technologies*.

[108] Ren C, Xu ZY, Sun S, Meksem K, Kahl G (2005). Genomic DNA libraries and physical mapping. *The Handbook of Plant Genome Mapping: Genetic and Physical Mapping*.

[109] Wu C, Xu Z, Zhang H-B, Meyers RA (2004). DNA libraries. *Encyclopedia of Molecular Cell Biology and Molecular Medicine*.

[110] Zhang H-B, Wu C (2001). BAC as tools for genome sequencing. *Plant Physiology and Biochemistry*.

[111] Zhang H-B, Woo S-S, Wing RA, Foster G, Twell D (1996). BAC, YAC and cosmid library construction. *Plant Gene Isolation: Principles and Practice*.

[112] Ioannou PA, Amemiya CT, Garnes J (1994). A new bacteriophage P1-derived vector for the propagation of large human DNA fragments. *Nature Genetics*.

[113] Shizuya H, Birren B, Kim U-J (1992). Cloning and stable maintenance of 300-kilobase-pair fragments of human DNA in Escherichia coli using an F-factor-based vector. *Proceedings of the National Academy of Sciences of the United States of America*.

[114] Chang Y-L, Tao Q, Scheuring C, Meksem K, Zhang H-B (2001). An integrated map of *Arabidopsis thaliana* for functional analysis of its genome sequence. *Genetics*.

[115] Hoskins RA, Nelson CR, Berman BP (2000). A BAC-based physical map of the major autosomes of *Drosophila melanogaster*. *Science*.

[116] International Human Genome Mapping Consortium (2001). A physical map of the human genome. *Nature*.

[117] Li Y, Uhm T, Ren C (2007). A plant-transformation-competent BIBAC/BAC-based map of rice for functional analysis and genetic engineering of its genomic sequence. *Genome*.

[118] Marra M, Kucaba T, Sekhon M (1999). A map for sequence analysis of the *Arabidopsis thaliana* genome. *Nature Genetics*.

[119] Mozo T, Dewar K, Dunn P (1999). A complete BAC-based physical map of the *Arabidopsis thaliana* genome. *Nature Genetics*.

[120] Ren C, Lee M-K, Yan B (2003). A BAC-based physical map of the chicken genome. *Genome Research*.

[121] Tao Q, Chang Y-L, Wang J (2001). Bacterial artificial chromosome-based physical map of the rice genome constructed by restriction fingerprint analysis. *Genetics*.

[122] Wallis JW, Aerts J, Groenen MAM (2004). A physical map of the chicken genome. *Nature*.

[123] Wu C, Sun S, Lee M-K, Xu ZY, Ren C, Zhang H-B, Meksem K, Kahl G (2005). Whole genome physical mapping: an overview on methods for DNA fingerprinting. *The Handbook of Plant Genome Mapping: Genetic and Physical Mapping*.

[124] Wu C, Sun S, Nimmakayala P (2004). A BAC- and BIBAC-based physical map of the soybean genome. *Genome Research*.

[125] Xu Z, Berg MVD, Scheuring C (2005). Genome-wide physical mapping from large-insert clones by fingerprint analysis with capillary electrophoresis: a robust physical map of Penicillium chrysogenum. *Nucleic Acids Research*.

[126] Xu Z, Sun S, Covaleda L (2004). Genome physical mapping with large-insert bacterial clones by fingerprint analysis: methodologies, source clone genome coverage, and contig map quality. *Genomics*.

[127] Zhang H-B, Wing RA (1997). Physical mapping of the rice genome with BACs. *Plant Molecular Biology*.

[128] Zhang X, Scheuring C, Tripathy S (2006). An integrated BAC and genome sequence physical map of *Phytophthora sojae*. *Molecular Plant-Microbe Interactions*.

[129] Adams MD, Celniker SE, Holt RA (2000). The genome sequence of *Drosophila melanogaster*. *Science*.

[130] The Arabidopsis Genome Initiative (2000). Analysis of the genome sequence of the flowering plant *Arabidopsis thaliana*. *Nature*.

[131] International Human Genome Sequencing Consortium (2001). Initial sequencing and analysis of the human genome. *Nature*.

[132] Sasaki T (2005). The map-based sequence of the rice genome. *Nature*.

[133] Tyler BM, Tripathy S, Zhang X (2006). *Phytophthora* genome sequences uncover evolutionary origins and mechanisms of pathogenesis. *Science*.

[134] Zhang H-B, Kole C, Abbott A (2007). Map-based cloning of genes and QTLs. *Plant Molecular Mapping and Breeding*.

[135] Chen M, Sanmiguel P, de Oliveira AC (1997). Microcolinearity in *sh*2-homologous regions of the maize, rice, and sorghum genomes. *Proceedings of the National Academy of Sciences of the United States of America*.

[136] Patocchi A, Vinatzer BA, Gianfranceschi L (1999). Construction of a 550 kb BAC contig spanning the genomic region containing the apple scab resistance gene Vf. *Molecular and General Genetics*.

[137] Zwick MS, Islam-Faridi MN, Czeschin DG (1998). Physical mapping of the liguleless linkage group in sorghum bicolor using rice RFLP-selected sorghum BACs. *Genetics*.

[138] Basra AS, Malik CP (1984). Development of the cotton fiber. *International Review of Cytology*.

[139] Wilkins TA, Arpat AB (2005). The cotton fiber transcriptome. *Physiologia Plantarum*.

[140] Wu Y, Machado AC, White RG, Llewellyn DJ, Dennis ES (2006). Expression profiling identifies gene expressed early during lint fiber initiation in cotton. *Plant Cell Physiology*.

[141] Nelson WM, Bharti AK, Butler E (2005). Whole-genome validation of high-information-content fingerprinting. *Plant Physiology*.

[142] Wu C, Wang S, Zhang H-B (2006). Interactions among genomic structure, function, and evolution revealed by comprehensive analysis of the *Arabidopsis* genome. *Genomics*.

[143] Hawkins JS, Kim H, Nason JD, Wing RA, Wendel JF (2006). Differential lineage-specific amplification of transposable elements is responsible for genome size variation in *Gossypium*. *Genome Research*.

[144] Dreher K, Morris M, Khairallah M, Ribaut JM, Pandey S, Srinivasan G Is marker-assisted selection cost-effective compared to conventional plant breeding methods? The case of quality protein maize.

[145] Guo W, Zhang T, Shen X, Yu JZ, Kohel RJ (2003). Development of SCAR marker linked to a major QTL for high fiber strength and its usage in molecular-marker assisted selection in upland cotton. *Crop Science*.

[146] Zhang JF, Stewart JMcD (2001). Inheritance and genetic relationships of the D8 and D2-2 restorer genes for cotton cytoplasmic male sterility. *Crop Science*.

[147] Guo W, Zhang T, Pan J, Kohel RJ (1998). Identification of RAPD marker linked with fertility-restoring gene of cytoplasmic male sterile lines in upland cotton. *Chinese Science Bulletin*.

[148] Zhang J, Stewart JMcD (2004). Identification of molecular markers linked to the fertility restorer genes for CMS-D8 in cotton. *Crop Science*.

[149] He L, Du CG, Covaleda L (2004). Cloning, characterization, and evolution of the NBS-encoding resistance gene analogue family in polyploid cotton (*Gossypium hirsutm* L.). *Molecular Plant-Microbe Interaction*.

[150] Gardiner J, Schroeder S, Polacco ML (2004). Anchoring 9,371 maize expressed sequence tagged unigenes to the bacterial artificial chromosome contig map by two-dimensional overgo hybridization. *Plant Physiology*.

